# Concordance of Chest Radiography and Chest Computed Tomography Findings in Patients with Hematologic Malignancy and Invasive Mucormycosis: What Are the Prognostic Implications?

**DOI:** 10.3390/jof10100703

**Published:** 2024-10-09

**Authors:** Sebastian Wurster, Sung-Yeon Cho, Hazim Allos, Alexander Franklin, Dierdre B. Axell-House, Ying Jiang, Dimitrios P. Kontoyiannis

**Affiliations:** 1 Department of Infectious Diseases, Infection Control, and Employee Health, University of Texas MD Anderson Cancer Center, Houston, TX 77030, USA; stwurster@mdanderson.org (S.W.); scho3@mdanderson.org (S.-Y.C.); hallos@mdanderson.org (H.A.); alexander.franklin@bcm.edu (A.F.); yijiang@mdanderson.org (Y.J.); 2 Division of Infectious Diseases, Department of Internal Medicine, Vaccine Bio Research Institute, College of Medicine, The Catholic University of Korea, Seoul 06591, Republic of Korea; 3 Catholic Hematology Hospital, Seoul St. Mary’s Hospital, Seoul 06591, Republic of Korea; 4 Section of Infectious Diseases, Department of Medicine, Baylor College of Medicine, Houston, TX 77030, USA; 5 Division of Infectious Diseases, Houston Methodist Hospital and Houston Methodist Research Institute, Houston, TX 77030, USA; dierdre.axell-house@bcm.edu

**Keywords:** mucormycosis, lung infection, hematological malignancy, radiologic imaging, chest computed tomography, chest X-ray, mortality, prognosis, neutropenia

## Abstract

Invasive pulmonary mucormycosis (IPM) is a deadly opportunistic mold infection in patients with hematological malignancies (HM). Radiologic imaging is essential for its timely diagnosis. Here, we compared IPM lesions visualized by chest computed tomography (CCT) and chest X-ray (CXR) and determined the prognostic significance of discordant imaging. Therefore, we reviewed 44 consecutive HM patients with probable/proven IPM at MD Anderson Cancer Center in 2000–2020 who had concurrent CCT and CXR studies performed. All 44 patients had abnormal CCTs and 39 (89%) had anormal CXR findings at IPM diagnosis. However, only 26 patients (59%) showed CCT-matching IPM-suspicious lesions on CXR. Acute Physiology and Chronic Health Evaluation II score > 18 at IPM diagnosis and breakthrough infection to Mucorales-active antifungals were the only independent risk factors for 42-day and/or 84-day mortality. Absence of neutropenia at IPM diagnosis, neutrophil recovery in neutropenic patients, and surgical revision of mucormycosis lesions were protective factors. Although not reaching significance on multivariable analysis, visualization of CCT-matching lesions on CXR was associated with significantly increased 84-day mortality (log-rank test, *p* = 0.033), possibly as a surrogate of extensive lesions and tissue necrosis. This observation supports the exploration of radiologic lesion kinetics as a prognostic staging tool in IPM patients.

## 1. Introduction

Invasive pulmonary mucormycosis (IPM) is a severe and often deadly infection in patients with hematological malignancies (HMs), especially those with acute leukemia and/or recipients of allogenic hematopoietic cell transplants (HCTs) [1]. Historically, chest X-ray (CXR) has been the primary radiographic modality to diagnose pulmonary abnormalities and remains a widely used screening test in HM patients with signs of respiratory infections [2,3]. However, due to improved availability and better sensitivity, chest computed tomography (CCT) has become the imaging modality of choice for early diagnosis of pulmonary mycoses, including IPM [4]. Typical CXR and CCT findings of IPM include pulmonary nodules, masses, and consolidations, with or without a reverse halo sign, i.e., an area of ground glass opacity surrounded by consolidation [5,6,7,8]. However, atypical radiologic presentations of IPM are common, especially in non-neutropenic patients [5,6,7,8].

Comparing initial CXR and CCT findings offers the opportunity to evaluate the sensitivity of these imaging modalities and to explore the clinical significance of discordant imaging. We hypothesized that there is a spectrum of disease burden in IPM, and lesions visualized on CXR may be associated with worse clinical outcomes than those visualized only by CCT. Therefore, we herein studied host characteristics, clinical presentation, and outcomes in patients with HM and probable/proven IPM depending on the presence of CCT-matching IPM lesions on conventional chest radiography.

## 2. Methods

### 2.1. Database and Chart Review

We utilized a previously curated database [9] of 103 adult patients with HM who developed probable or proven IPM (EORTC/MSG consensus criteria) [10] at the University of Texas MD Anderson Cancer Center, a tertiary oncologic center in Houston, Texas, between April 2000 and April 2020. The present analysis was restricted to patients who had IPM or disseminated mucormycosis with lung involvement and had both CXR and CCT performed within 5 days of each other and within 7 days of symptom onset or IPM diagnosis (i.e., collection date of first positive culture or histopathology). In patients with repeated imaging studies, the CCT closest to the time of IPM diagnosis and the CXR closest to the time of the reference CCT were reviewed.

### 2.2. Adjudication of Radiologic Images

**Figure 1:**
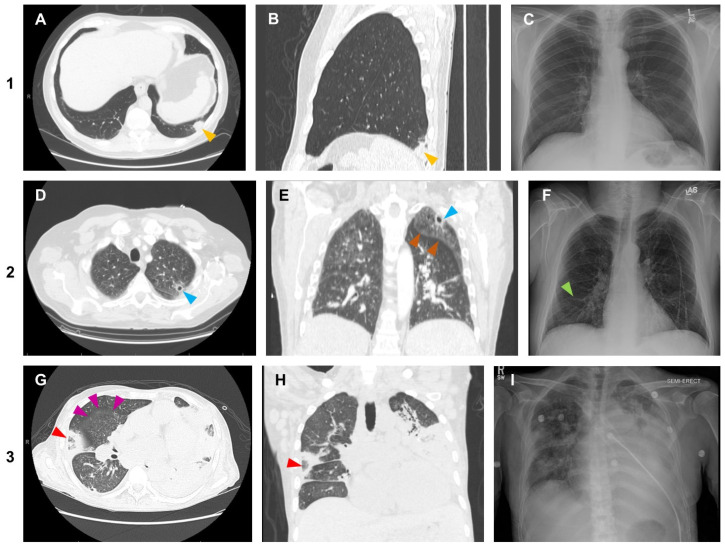
Representative radiologic images from patients with (1) an IPM-suspicious CCT but normal CXR, (2) IPM-suspicious CCT with abnormal but discordant CXR, and (3) matching IPM-suspicious lesions on CCT and CXR. Patient 1: (**A**,**B**) CCTs showing a 2 cm solid nodule in the left lower lobe (yellow arrowhead). Other CCT images not included in this figure revealed ground glass opacities with nodules in the right lower lobe, indicative of multifocal infection. (**C**) Largely normal CXR without signs of pneumonia. Patient 2: (**D**,**E**) CCTs showing bilateral ill-defined ground glass opacities (brown arrowheads) and nodules predominating in the upper lobes, one of which is cavitating (blue arrowheads). (**F**) CXR not revealing the lesions seen on CCT but showing linear opacities in the right middle lobe (green arrowhead) and slightly increased opacity in the right apex. Patient 3: (**G**,**H**) CCT showing bilateral pneumonia with multifocal consolidation and opacities (e.g., in the area highlighted with purple arrowheads) and nodules/consolidations with reverse halo morphology (red arrowheads). (**I**) CXR showing bilateral airspace disease with numerous opacities and nodules that are consistent with the CCT findings. Abbreviations: CCT = chest computed tomography; CXR = chest X-ray; IPM = invasive pulmonary mucormycosis.

Patients were adjudicated as having matching CCT and CXR if both modalities showed at least one matching IPM-suspicious lesion (nodule, consolidation, or mass-like lesion) of the same type and at the same anatomic location. Determinations were made independently by two Infectious Diseases attendings (A.F. and S.-Y.C.), taking into account the notes provided by the radiologists in the medical record system. Discrepancies were reviewed and adjudicated by a third Infectious Diseases faculty member (S.W.). Representative radiologic images from patients with (1) an IPM-suspicious CCT but normal CXR, (2) IPM-suspicious CCT with abnormal but discordant CXR, and (3) matching IPM-suspicious lesions on CCT and CXR are shown in Figure 1.

### 2.3. Definitions

Neutropenia and lymphopenia were defined as an absolute neutrophil or lymphocyte count < 500/µL. Significant glucocorticosteroid (GCS) use was defined as a cumulative dose > 600 mg of prednisolone equivalent during the month prior to MCR diagnosis. Hypoalbuminemia was defined as a serum albumin level of <3 mg/dL. Breakthrough IPM was defined as receipt of isavuconazole, posaconazole, or liposomal amphotericin B at the time of IPM diagnosis [9].

### 2.4. Statistical Analyses

We performed two comparisons: (i) patients with CCT-matching versus discordant CXR lesions and (ii) patients who survived until day 42 and 84 after IPM symptom onset versus those who expired. Categorical variables were compared using chi-squared or Fisher’s exact test, as appropriate. Continuous variables were compared using Wilcoxon’s rank sum test. Survival curves were compiled using the Kaplan–Meier method and compared using the Mantel–Cox log-rank test. Additionally, multivariable Cox’s proportional hazard models were used to identify independent predictors of 42-day and 84-day survival. All tests were two-sided. *p*-values < 0.05 were considered significant. Data analysis and visualization was performed using Microsoft Office Excel 365 (Microsoft Corporation, Redmond, WA, USA), Prism v10 (GraphPad Software, La Jolla, CA, USA), and SAS version 9.4 (SAS Institute Inc., Cary, NC, USA).

## 3. Results

### 3.1. Demographics and Clinical Characteristics of Our IPM Cohort

We identified 44 HM patients with probable/proven IPM who had concurrent CCT and CXR studies performed within the time frame specified in Section 2.1 (Table 1). Thirty-one patients (70%) were male, and the median age was 54 years (range: 23–76). 

Most patients had leukemia or myelodysplastic syndrome (86%) and had active malignancy (84%). Twenty-one patients (48%) had received an HCT and most of them had graft-versus-host disease (18/21, 86%). Over two-thirds of patients had neutropenia (70%) and/or lymphopenia (73%). Hypoalbuminemia (91%) and ongoing immunosuppressive therapy (70%), including significant GCS therapy (32%), were also common. 

*Rhizopus* was the commonest causative Mucorales genus (52%). Fifty-nine percent of patients had additional extrapulmonary mucormycosis manifestations, including sinusitis (27%). Of note, 12 patients (27%) had breakthrough IPM to Mucorales-active antifungal prophylaxis or therapy. All patients received appropriate antifungal therapy, most commonly liposomal amphotericin B (80%). Additionally, 10 patients (23%) received surgical IPM therapy.

### 3.2. Radiologic Findings and Concordance between CCT and CXR

All 44 patients had an abnormal CCT (Table 2). The commonest IPM-suggestive CCT findings were consolidations (*n* = 31, 70%), pleural effusions (*n* = 31, 70%), nodules with or without halo sign (*n* = 26, 59%), ground-glass opacities (*n* = 16, 36%), and reverse halo or cavitary nodules (*n* = 12, 27%) (Table 2). Thirty-nine out of the forty-four patients (89%) had abnormal findings on CXR, most commonly consolidations (*n* = 28, 64%) and pleural effusions (*n* = 19, 43%) (Table 2). Other IPM-suggestive findings, i.e., ground-glass opacities (*n* = 7, 16%), reverse halo or cavitary nodules (*n* = 7, 16%), and nodules with or without halo sign (*n* = 6, 14%; *p* < 0.001 vs. CCT), were less common on CXR than on CCT (Table 2).

**Figure 2:**
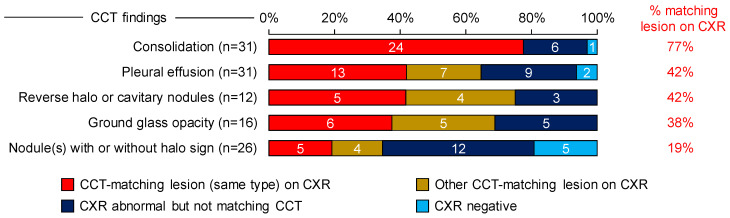
Concordance of CCT and CXR findings. Numbers of patients by type of mucormycosis-suspicious CCT finding, subdivided by concordance of lesions visualized on CXR. Percentages on the right represent the proportion of patients with matching CXR lesions of the same type among those who showed the respective CCT feature. Abbreviations: CCT = chest computed tomography; CXR = chest X-ray.

Interestingly, only 26 patients (59%) had a CCT-matching lesion on CXR, whereas 13 patients (30%) had abnormal CXR findings not matching the CCT, and 5 patients (11%) had a negative CXR. Twenty-four out of thirty-one patients (77%) with consolidation on CCT had matching CXR findings (Figure 2). In contrast, pleural effusions (13/31, 42%), halo/cavitary nodules (5/12, 42%), ground glass opacities (6/16, 38%), and nodules (5/26, 19%) on CCT were less commonly associated with matching CXR findings (9/26, 35%, Figure 2).

### 3.3. Comparison of Clinical Characteristics in Patients with and without CCT-Matching CXR

Given the small number of patients without abnormal CXR findings (*n* = 5), we subsequently focused on a two-group comparison between patients with CCT-matching CXR findings (*n* = 26) and those with discordant imaging (*n* = 18), i.e., patients with either negative CXR or non-matching lesions. No significant differences in demographics, host factors, or causative Mucorales genera were found between these two groups (Table 1).

Because non-neutropenic patients with IPM have a higher propensity to present with atypical radiologic findings [5,6,7], we additionally compared underlying host factors and infection characteristics specifically in patients who were neutropenic at the time of IPM diagnosis. As was the case for the overall cohort, no significant differences were found between neutropenic IPM patients with CCT-matching CXR findings and those with discordant imaging (Table S1).

### 3.4. Comparison of IPM Outcomes between Patients with and without CCT-Matching CXR

**Figure 3:**
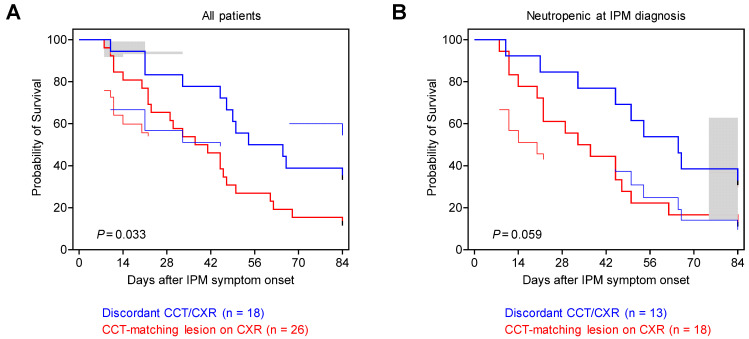
CCT-matching lesions on CXR are associated with worse 84-day mortality outcomes in patients with hematological malignancies and IPM. Kaplan–Meier survival curves for days 0–84 after IPM symptom onset in all patients included in this study (**A**, *n* = 44) or only those with neutropenia (absolute neutrophil count < 500) at IPM diagnosis (**B**, *n* = 31), subdivided by presence or absence of CCT-matching IPM lesions on CXR. Error bands denote 95% confidence intervals. Mantel–Cox log-rank test. Abbreviations: CCT = chest computed tomography; CXR = chest X-ray; IPM = invasive pulmonary mucormycosis.

Although infection severity at the time of IPM diagnosis was comparable between patients with matching CXR lesions and those with discordant imaging (median Acute Physiology and Chronic Health Evaluation [APACHE II] scores, 16 versus 15, *p* = 0.792, Table 1), we found several signals of worse prognosis in IPM patients with CCT-matching CXR lesions. Specifically, median survival periods after both symptom onset (37 vs. 52 days, *p* = 0.051) and IPM diagnosis (28 vs. 37 days, *p* = 0.168) tended to be shorter in patients with CCT-matching CXR lesions than in those with discordant imaging (Table 3). Consequently, those with CCT-matching visualization of IPM lesions on CXR had significantly higher 42-day mortality (54% versus 22%, *p* = 0.036; overall: 41%) and a trend toward increased 84-day mortality (88% versus 67%, *p* = 0.128; overall: 80%) after IPM symptom onset compared to patients with discordant imaging (Table 3). This trend was further corroborated by 84-day survival curve analysis after IPM symptom onset (*p* = 0.033, Figure 3A) and largely persisted when restricting the analysis to the 31 patients who were neutropenic at IPM diagnosis (*p* = 0.059, Figure 3B).

### 3.5. Predictors of 42- and 84-Day All-Cause Mortality after IPM Symptom Onset

On univariate analysis, variables significantly associated with increased 42-day all-cause mortality after IPM symptom onset included lack of neutrophil recovery in neutropenic patients (*p* < 0.001), lack of surgical therapy of IPM (*p* = 0.003), higher APACHE II scores at IPM diagnosis (*p* = 0.002), and ICU admission at any time during IPM therapy (*p* = 0.003, Table 4). Likewise, higher APACHE II scores at IPM diagnosis (*p* < 0.001) and lack of surgical therapy of IPM (*p* = 0.018) were also associated with increased 84-day all-cause mortality after IPM symptom onset. Additionally, breakthrough IPM to Mucorales-active antifungal prophylaxis/therapy was associated with universal death by day 84 after symptom onset (*p* = 0.047, Table 4).

On multivariable analysis, APACHE II score > 18 at IPM diagnosis (adjusted hazard ratio [aHR] 3.69/2.10; 95% confidence interval [CI] 1.31–10.37/1.004–4.39; *p* = 0.013/0.049 for 42- and 84-day mortality, respectively) was a significant independent risk factor for poor IPM outcomes (Table 5 (A,B)). Additionally, breakthrough IPM to Mucorales-active antifungals (aHR 3.13; 95% CI 1.03–9.47; *p* = 0.044) was an independent risk factor for 42-day mortality (Table 5 (A)). Inversely, absence of neutropenia at IPM diagnosis (only significant for 84-day outcome; aHR 0.40; 95% CI 0.17–0.97; *p* = 0.043), neutrophil recovery in patients with neutropenia at IPM diagnosis (aHR 0.08/0.25; 95% CI 0.02–0.40/0.10–0.58; *p* = 0.002/0.001 for 42- and 84-day mortality, respectively), and surgical therapy of mucormycosis lesions (only significant for 84-day outcome; aHR 0.34; 95% CI 0.13–0.93; *p* = 0.035) were significant independent predictors of favorable survival outcomes (Table 5 (A,B)). Although significant on univariate analysis, presence of CCT-matching CXR lesions was not identified as a significant independent predictor of IPM outcomes in our multivariable models.

## 4. Discussion

While CXR is often performed as a screening test in HM patients with signs of respiratory infections, CCT imaging has become widely available, remains indispensable due to its higher sensitivity for subtle lesions, and can reveal evidence of IPM earlier than CXR [11,12]. Although abnormal CXRs were common (89%) in our severely immunocompromised cohort of HM patients with IPM, a negative CXR does not preclude IPM. This aligns with prior evidence suggesting that up to a quarter of IPM-suspicious CCT findings are not uncovered by the initial CXR [13]. Notably, three out of the five IPM patients with negative CXR in our study were neutropenic, underscoring that negative CXR imaging may be encountered even in neutropenic patients. In addition to its greater overall sensitivity, CCT more commonly revealed signs highly suggestive of IPM (e.g., reverse halo sign), whereas CXR findings were partially unspecific (Table 2). 

Given the small number of IPM patients without abnormal CXR findings, we subsequently focused on the clinical significance of CCT-matching CXR findings versus discordant imaging (i.e., either negative or non-matching CXR findings). Although significance was not reached on multivariable analysis, CCT-matching visualization of mucormycosis-suspicious lesions on CXR at the time of IPM diagnosis was associated with increased 42- and 84-day all-cause mortality. Of note, this trend persisted when restricting the analysis to neutropenic patients and was not confounded by differences in causative Mucorales genus, breakthrough infection status to Mucorales-active antifungals, ongoing immunosuppressive therapies, or any other studied host factors (Table 1). Although our small dataset does not allow for us to preclude all conceivable confounders (e.g., co-infections), we hypothesize that CCT-matching visualization of IPM on CXR is a surrogate of more extensive lesions, possibly reflective of high fungal burden and/or surrounding tissue necrosis. 

It has been notoriously difficult to gauge the disease burden in patients with IPM due to a lack of reliable quantitative biomarkers and unreliability of patient-reported symptom burden. Therefore, our findings would support the exploration of quantitative analysis of lesion size/volume on radiologic imaging as a prognostic staging tool in IPM patients to identify those who might benefit from more aggressive management. In addition to potential bias due to changes in radiologic imaging technology, quality, and protocols during the 20-year review period, our dataset was too small for such analyses and would have lacked the statistical power to properly dissect granular data (i.e., quantitative lesion kinetics for various radiologic features) in the setting of dynamic changes in the net state of immunosuppression. Therefore, such analyses would be performed more suitably on prospectively enrolled contemporary multi-center cohorts or image repositories from clinical mycology trials obtained in a more standardized manner within a shorter period. Although detailed quantitative analyses are yet to be performed, multifocal lesions on CCT have been associated with heightened mortality of IPM [6]. Likewise, extensive lesion volume was a strong predictor of treatment failure and mortality in patients with invasive pulmonary aspergillosis [14].

While data specifically for mold pneumonias are scarce, the epidemiology and significance of CCT-discordant CXR findings have been previously studied in other settings. For instance, only 43.5% of adult emergency department patients with cardiorespiratory symptoms and opacities on CCT had matching opacities on CXR, likely due to enhanced CCT-based visualization of small opacities or those located in the lung bases or lingula [15]. Additionally, several prior studies reported frequent discordance of CXR and CCT findings in patients with community-acquired pneumonia (CAP) [16,17]. Specifically, Upchurch and colleagues found differences in the pathogen spectrum but comparable clinical outcomes of CAP patients with and without CCT-matching CXR findings [17]. However, the study excluded patients with HM and many other immunocompromising conditions. 

The devastating outcomes of IPM in our cohort of HM patients, with an 84-day all-cause mortality rate of 80% despite aggressive diagnostic work-up and initiation of appropriate antifungal therapy in all patients (Table 1), underscores the urgent need for improved IPM management. In particular, the median duration of 10.5 days from symptom onset until definitive culture- or histopathology-driven IPM diagnosis in our cohort is a painful reminder of the urgent need for improved early culture-independent diagnostic modalities such as quantitative polymerase chain reaction [18]. Furthermore, the significant impact of persistent host immune failure (i.e., unrecovered neutropenia, Table 5) on IPM outcomes underscores the need for adjunctive host-targeted therapies [19]. Moreover, our multivariable Cox regression analysis corroborated that breakthrough IPM to Mucorales-active antifungals portrays poor prognosis in patients with HM, aligning with our previous observation in a larger and more heterogenous cohort of mucormycosis patients, including those with non-pulmonary manifestations [9]. Potential hypotheses for this observation, including fungal plasticity and increased Mucoralean virulence after subinhibitory exposures to some triazoles, have been discussed elsewhere [9,20].

This study has several limitations: Firstly, due to the restrictive inclusion criteria, numbers of patients were low despite the long review period. Therefore, our analyses have limited power and might not have captured all potential confounders. Secondly, we did not collect data regarding the specific anatomic location of the lesions within the lungs or data regarding the number or size of IPM lesions on either CCT or CXR. As this study focused on the concordance of initial imaging, we also did not analyze any follow-up imaging studies. Thirdly, this study was based on a retrospective review of data collected for routine clinical diagnostics in a non-blinded setting and thus subject to both inter-observer variability and potential bias of the radiology notes due to the evaluator’s knowledge of other clinical and radiologic findings. Lastly, given the broad use of triazole prophylaxis in our high-risk HM patients, all patients developed IPM while receiving mold-active antifungals, including those with poor Mucorales activity, such as echinocandins. Therefore, our findings might not be transferable to non-oncologic populations at risk for mucormycosis who commonly do not receive antifungal prophylaxis, e.g., patients with uncontrolled diabetes, high-dose GCS use, and/or COVID-19. 

## 5. Conclusions

Although abnormal CXR findings were common (89%) in our IPM cohort, less than two thirds of patients had CCT-matching lesions on contemporary CXR imaging. CCT-matching visualization of mucormycosis-suspicious lesions on CXR at the time of IPM diagnosis was associated with poor outcomes, possibly as a surrogate of extensive lesions, high fungal burden, and tissue necrosis. Future multi-center studies in larger and more diverse cohorts, along with quantitative studies of pulmonary lesion volume, are warranted to corroborate our findings and to elucidate whether IPM patients with more extensive lesions on radiologic imaging would benefit from more aggressive clinical management.

## Figures and Tables

**Table 1:** Univariate comparison of host characteristics, IPM manifestations, and therapy in patients with and without CCT-matching lesions on CXR. Unless indicated otherwise, numbers of patients and percentages (in parentheses) are provided.

Characteristics	All Patients	CCT-Matching Lesions on CXR		*p*-Value
	*n* = 44	Yes (*n* = 26)	No (*n* = 18)	
**Demographics**				
Age (years), median (range)	54 (23–76)	59 (23–76)	50 (23–67)	0.104
Sex, male	31 (70)	16 (62)	15 (83)	0.119
Race				0.452
White	34 (77)	19 (73)	15 (83)	
Black	6 (14)	5 (19)	1 (6)	
Hispanic	3 (7)	2 (8)	1 (6)	
Asian	1 (2)	0 (0)	1 (6)	
**Underlying malignancy and other risk factors**				
Type of malignancy				0.375
Leukemia/myelodysplastic syndrome	38 (86)	21 (81)	17 (94)	
Lymphoma/myeloma	6 (14)	5 (19)	1 (6)	
Malignancy status				0.103
Active	37 (84)	24 (92)	13 (72)	
Remission	7 (16)	2 (8)	5 (28)	
Allogenic HCT	21 (48)	11 (42)	10 (56)	0.387
Graft-versus-host disease	18/21 (86)	9/11 (82)	9/10 (90)	>0.999
ANC at IPM diagnosis, median (IQR)	0 (0–1030)	10 (0–910)	0 (0–1300)	0.877
ANC < 500/µL at IPM diagnosis	31 (70)	18 (69)	13 (72)	0.831
ANC recovery by day +42 (or death)	17/31 (55)	8/18 (44)	9/13 (69)	0.171
Duration of ANC < 500/µL at IPM diagnosis (days), median (IQR)	13 (0–30)	11 (0–37)	14 (0–22)	0.866
ALC at IPM diagnosis, median (IQR)	90 (0–520)	135 (0–1110)	35 (0–360)	0.214
ALC < 500/µL at IPM diagnosis	32 (73)	18 (69)	14 (78)	0.733
Ongoing immunosuppressive therapy	31 (70)	19 (73)	12 (67)	0.647
Significant GCS use	14 (32)	8 (31)	6 (33)	0.858
Hypoalbuminemia	40 (91)	23 (88)	17 (94)	0.634
Breakthrough IPM to Mucorales-active antifungals	12 (27)	8 (31)	4 (22)	0.733
**IPM presentation and therapy**				
Mucormycosis classification				0.548
Proven	27 (61)	15 (58)	12 (67)	
Probable	17 (39)	11 (42)	6 (33)	
Any extrapulmonary manifestation	26 (59)	15 (58)	11 (61)	0.821
Sinusitis	12 (27) ^X^	6 (23)	6 (33) ^X^	0.506
Other extrapulmonary manifestation	15 (34) ^X^	9 (35)	6 (33) ^X^	0.930
Causative genus				0.414
*Rhizopus*	23 (52)	16 (62)	7 (39)	
*Mucor*	8 (18)	3 (12)	5 (28)	
*Rhizomucor*	7 (16)	4 (15)	3 (17)	
*Cunninghamella*	5 (11)	3 (12)	2 (11)	
*Absidia*	1 (2)	0 (0)	1 (6)	
APACHE II score at IPM diagnosis, median (IQR)	15 (14–19)	16 (14–19)	15 (13–19)	0.792
Initial antifungal therapy				0.666
Liposomal amphotericin B	35 (80)	21 (81)	14 (78)	
Posaconazole	8 (18)	5 (19)	3 (17)	
Isavuconazole	1 (2)	0 (0)	1 (6)	
Surgical therapy of IPM	10 (23)	4 (15)	6 (33)	0.273
ICU at diagnosis	5 (11)	4 (15)	1 (6)	0.634
ICU admission at any time during therapy of IPM	25 (57)	17 (65)	8 (44)	0.168

^X^ One patient had both. Abbreviations: ALC = absolute lymphocyte count; ANC = absolute neutrophil count; APACHE II = Acute Physiology and Chronic Health Evaluation II score; CCT = chest computed tomography; CXR = chest X-ray; GCS = glucocorticosteroids; HCT = hematopoietic cell transplant; ICU = intensive care unit; IPM = invasive pulmonary mucormycosis; IQR = inter-quartile range.

**Table 2:** Frequency of (overlapping) mucormycosis-suspicious findings on CCT and CXR.

Finding	CCT	CXR	*p*-Value
Any abnormal finding	44 (100)	39 (89)	0.055
Consolidation	31 (70)	28 (64)	0.496
Pleural effusion	31 (70)	19 (43)	0.017
Nodule(s) with or without halo sign	26 (59)	6 (14)	<0.001
Ground-glass opacity	16 (36)	7 (16)	0.051
Reverse halo or cavitary nodules	12 (27)	7 (16)	0.195
Only unspecific CXR findings ^#^	Not applicable	4 (9)	Not applicable

^#^ e.g., heterogenous or non-specific patchy opacities. Abbreviations: CCT = chest computed tomography; CXR = chest X-ray.

**Table 3:** Comparison of outcomes in IPM patients with and without CCT-matching lesions on CXR.

Outcome	All Patients	CCT-Matching Lesions on CXR		*p*-Value
	*n* = 44	Yes (*n* = 26)	No (*n* = 18)	
Days from IPM symptom onset to death, median (IQR)	45 (22–65)	37 (22–50)	52 (39–87)	0.051
Days from IPM diagnosis * to death, median (IQR)	31 (7–50)	28 (7–44)	37 (17–68)	0.168
Died within 42 days of IPM symptom onset, n (%)	18 (41)	14 (54)	4 (22)	0.036
Died within 84 days of IPM symptom onset, n (%)	35 (80)	23 (88)	12 (67)	0.128

* Collection date of first positive culture or histopathology, which occurred at a median of 10.5 days after IPM symptom onset. Abbreviations: CCT = chest computed tomography; CXR = chest X-ray; IPM = invasive pulmonary mucormycosis; IQR = inter-quartile range.

**Table 4:** Univariate analysis of variables associated with 42- and 84-day all-cause mortality after IPM symptom onset. Unless indicated otherwise, numbers of patients and percentages (in parentheses) are provided.

Characteristics	Day 42 Outcome ^$^			Day 84 Outcome ^$^		
	Survived (*n* = 26)	Died (*n* = 18)	*p*-Value	Survived (*n* = 9)	Died (*n* = 35)	*p*-Value
**Demographics**						
Age (years), median (range)	50 (23–76)	60 (23–75)	0.056	41 (23–67)	57 (23–76)	0.051
Sex, male	19 (73)	12 (67)	0.647	7 (78)	24 (69)	0.703
Race			0.838			0.710
White	20 (77)	14 (78)		7 (78)	27 (77)	
Black	4 (15)	2 (11)		2 (22)	4 (11)	
Hispanic	1 (4)	2 (11)		0 (0)	3 (9)	
Asian	1 (4)	0 (0)		0 (0)	1 (3)	
**Underlying malignancy and other risk factors**						
Type of malignancy			0.208			>0.999
Leukemia/myelodysplastic syndrome	24 (92)	14 (78)		8 (89)	30 (86)	
Lymphoma/myeloma	2 (8)	4 (22)		1 (11)	5 (14)	
Malignancy status			>0.999			0.138
Active	22 (85)	15 (83)		6 (67)	31 (89)	
Remission	4 (15)	3 (17)		3 (33)	4 (11)	
Allogenic HCT	15 (58)	6 (33)	0.112	6 (67)	15 (43)	0.272
Graft-versus-host disease	12/15 (80)	6/6 (100)	0.526	5/6 (83)	13/15 (87)	>0.999
ANC at IPM diagnosis, median (IQR)	0 (0–4370)	10 (0–910)	0.857	0 (0–4780)	0 (0–910)	0.695
ANC < 500/µL at IPM diagnosis	18 (69)	13 (72)	0.831	6 (67)	25 (71)	>0.999
ANC recovery by day +42 (or death)	15/18 (83)	2/13 (15)	<0.001	5/6 (83)	12/25 (48)	0.185
Duration of ANC < 500/µL at IPM diagnosis (days), median (IQR)	11 (0–22)	21 (7–46)	0.147	8 (0–11)	18 (3–37)	0.108
ALC at IPM diagnosis, median (IQR)	185 (0–440)	70 (0–660)	0.863	190 (0–300)	90 (0–530)	0.776
ALC < 500/µL at IPM diagnosis	20 (77)	12 (67)	0.506	7 (78)	25 (71)	>0.999
Ongoing immunosuppressive therapy	19 (73)	12 (67)	0.647	7 (78)	24 (69)	0.703
Significant GCS use	8 (31)	6 (33)	0.858	3 (33)	11 (31)	>0.999
Hypoalbuminemia	23 (88)	17 (94)	0.634	9 (100)	31 (89)	0.566
Breakthrough IPM to Mucorales-active antifungals	5 (19)	7 (39)	0.183	0 (0)	12 (34)	0.047
**IPM presentation and therapy**						
Mucormycosis classification			0.510			>0.999
Proven	17 (65)	10 (56)		6 (67)	21 (60)	
Probable	9 (35)	8 (44)		3 (33)	14 (40)	
Any extrapulmonary manifestation	18 (69)	8 (44)	0.100	6 (67)	20 (57)	0.716
Sinusitis	8 (31) ^X^	4 (22)	0.733	2 (22)	10 (29) ^X^	>0.999
Other extrapulmonary manifestation	11 (42) ^X^	4 (22)	0.167	4 (44)	11 (31) ^X^	0.464
CCT-matching lesion on CXR	12 (46)	14 (78)	0.061	3 (33)	23 (66)	0.128
Causative genus			0.896			0.874
*Rhizopus*	12 (46)	11 (61)		4 (44)	19 (54)	
*Rhizomucor*	5 (19)	2 (11)		2 (22)	5 (14)	
*Mucor*	5 (19)	3 (17)		2 (22)	6 (17)	
*Cunninghamella*	3 (12)	2 (11)		1 (11)	4 (11)	
*Absidia*	1 (4)	0 (0)		0 (0)	1 (4)	
APACHE II score at IPM diagnosis, median (IQR)	14 (12–16)	18 (15–20)	0.002	12 (9–14)	16 (14–19)	<0.001
Initial antifungal therapy			0.539			>0.999
Liposomal amphotericin B	19 (73)	16 (89)		7 (78)	28 (80)	
Posaconazole	6 (23)	2 (11)		2 (22)	6 (17)	
Isavuconazole	1 (4)	0 (0)		0 (0)	1 (3)	
Surgical therapy of IPM	10 (38)	0 (0)	0.003	5 (56)	5 (14)	0.018
ICU at diagnosis	1 (4)	4 (22)	0.142	0 (0)	5 (14)	0.566
ICU admission at any time during therapy of IPM	10 (38)	15 (83)	0.003	3 (33)	22 (63)	0.144

^$^ After onset of IPM symptoms. ^X^ One patient had both. Abbreviations: ALC = absolute lymphocyte count; ANC = absolute neutrophil count; APACHE II = Acute Physiology and Chronic Health Evaluation II score; CCT = chest computed tomography; CXR = chest X-ray; GCS = glucocorticosteroids; HCT = hematopoietic cell transplant; ICU = intensive care unit; IPM = invasive pulmonary mucormycosis; IQR = inter-quartile range.

**Table 5:** Independent predictors of 42- and 84-day all-cause mortality after IPM symptom onset.

(A) Predictors of 42-day mortality after IPM symptom onset	Adjusted HR	95% CI	*p*-Value
APACHE II score ≥ 18 at IPM diagnosis	3.69	1.31–10.37	0.013
Neutropenia (ANC < 500/µL) at IPM diagnosis			0.004
Yes, without recovery	Reference		
Yes, with recovery	0.08	0.02–0.40	0.002
No	0.38	0.13–1.16	0.089
Breakthrough IPM to Mucorales-active antifungals	3.13	1.03–9.47	0.044
**(B) Predictors of 84-day mortality after IPM symptom onset**	**Adjusted HR**	**95% CI**	***p*-value**
APACHE II score ≥ 18 at IPM diagnosis	2.10	1.004–4.39	0.049
Neutropenia (ANC < 500/µL) at IPM diagnosis			0.006
Yes, without recovery	Reference		
Yes, with recovery	0.25	0.10–0.58	0.001
No	0.40	0.17–0.97	0.043
Surgical therapy of IPM	0.34	0.13–0.93	0.035

Abbreviations: ANC = absolute neutrophil count; APACHE II = Acute Physiology and Chronic Health Evaluation II; CI = confidence interval; HR = hazard ratio; IPM = invasive pulmonary mucormycosis.

## Data Availability

Data are contained within the article and supplement.

## Supplementary Materials

The following supporting information can be downloaded at: https://www.mdpi.com/article/10.3390/jof10100703/s1, Table S1: Univariate comparison of host characteristics, IPM manifestations, and therapy in neutropenic patients with and without CCT-matching lesions on CXR.
