# Supplementary material for: Concordance of Chest Radiography and Chest Computed Tomography Findings in Patients with Hematologic Malignancy and Invasive Mucormycosis: What Are the Prognostic Implications?
Source: J Fungi (Basel). 2024 Oct 9;10(10):703. doi: 10.3390/jof10100703 (PMC11508256; doi:10.3390/jof10100703)
Supplement: Supplementary file 1 [file jof-10-00703-s001.zip › jof-3192418-supplementary.pdf]

Concordance of Chest Radiography and Chest Computed Tomography Findings in Patients with Hematologic Malignancy and Invasive Mucormycosis: What Are the Prognostic Implications?

Supplementary Data

Table S1. Univariate comparison of host characteristics, IPM manifestations and therapy in neutropenic patients with and without CCT-matching lesions on CXR. Unless indicated otherwise, numbers of patients and percentages (in parentheses) are provided.

Characteristics	All patients n=31	CCT-matching lesions on CXR Yes (n=18)	No (n=13)	*P*-value
**Demographics**				
Age (years), median (range)	56 (23-75)	61 (23-75)	51 (23-67)	0.229
Sex, male	22 (71)	11 (61)	11 (85)	0.237
Race				0.136
White	24 (77)	13 (72)	11 (85)	
Black	4 (13)	4 (22)	0 (0)	
Hispanic	2 (6)	1 (6)	1 (8)	
Asian	1 (3)	0 (0)	1 (8)	
**Underlying malignancy and other risk factors**				
Type of malignancy				>0.999
Leukemia/myelodysplastic syndrome	30 (97)	17 (94)	13 (100)	
Lymphoma/myeloma	1 (3)	1 (6)	0 (0)	
Malignancy status				0.419
Active	30 (97)	18 (100)	12 (92)	
Remission	1 (3)	0 (0)	1 (6)	
Allogenic HCT	11 (35)	5 (28)	6 (46)	0.449
Graft-versus-host disease	8/11 (73)	3/5 (60)	5/6 (83)	0.546
ANC at IPM diagnosis, median (IQR)	0 (0-20)	0 (0-20)	0 (0-0)	0.795
ANC < 500/µL at IPM diagnosis	Part of the cohort definition			
ANC recovery by day +42 (or death)	17 (55)	8 (44)	9 (69)	0.171
Duration of ANC <500/µL at IPM diagnosis (days), median (IQR)	20 (11-47)	22 (10-47)	16 (14-36)	>0.999
ALC at IPM diagnosis, median (IQR)	0 (0-360)	60 (0-440)	0 (0-300)	0.246
ALC < 500/µL at IPM diagnosis	25 (81)	14 (78)	11 (85)	>0.999
Ongoing immunosuppressive therapy	22 (71)	13 (72)	9 (69)	>0.999
Significant glucocorticosteroid use	4 (13)	1 (6)	3 (23)	0.284
Hypoalbuminemia	27 (87)	15 (83)	12 (92)	0.621
Breakthrough IPM to Mucorales-active antifungals	10 (32)	8 (44)	2 (15)	0.129
**IPM presentation and therapy**				
Mucormycosis classification				0.284
Proven	18 (58)	9 (50)	9 (69)	
Probable	13 (42)	9 (50)	4 (31)	
Any extrapulmonary manifestation	17 (55)	10 (56)	7 (54)	0.925
Sinusitis	8 (26) [X]	4 (22)	4 (31) [X]	0.689
Other extrapulmonary manifestation	10 (32) [X]	6 (33)	4 (31) [X]	>0.999
Causative genus				0.322
Rhizopus	15 (48)	11 (61)	4 (31)	
Mucor	8 (26)	3 (17)	5 (38)	
Rhizomucor	5 (16)	3 (17)	2 (15)	
Cunninghamella	2 (6)	1 (6)	1 (8)	
Absidia	1 (3)	0 (0)	1 (8)	
APACHE II score at IPM diagnosis, median (IQR)	16 (14-19)	16 (14-18)	15 (14-19)	0.840
Antifungal therapy				0.628
Liposomal amphotericin B	24 (77)	14 (78)	10 (77)	
Posaconazole	6 (19)	4 (22)	2 (15)	
Isavuconazole	1 (3)	0 (0)	1 (8)	
Surgical therapy of IPM	5 (16)	2 (11)	3 (23)	0.625
ICU at diagnosis	3 (10)	2 (11)	1 (8)	>0.999
ICU admission at any time during therapy of IPM	17 (55)	11 (61)	6 (46)	0.409

[X] One patient had both.

Abbreviations: ALC = absolute lymphocyte count, ANC = absolute neutrophil count, APACHE II = Acute Physiology and Chronic Health Evaluation II score, CCT = chest computed tomography, CXR = chest x-ray, HCT = hematopoietic cell transplant, ICU= intensive care unit, IPM = invasive pulmonary mucormycosis, IQR = inter-quartile range.
